# Reply to: Predictive factors for high-flow nasal cannula failure in patients with acute viral bronchiolitis admitted to the pediatric intensive care unit

**DOI:** 10.62675/2965-2774.20260414

**Published:** 2026-04-16

**Authors:** Patrick Jacobsen Westphal, Cassiano Teixeira, João Ronaldo Mafalda Krauzer, Mirelle Hugo Bueno, Priscilla Alves Pereira, Sandro V. Hostyn, Marcela Doebber Vieira, Camila Durante, Cristiane Bündchen

**Affiliations:** 1 Hospital Moinhos de Vento Porto Alegre RS Brazil Hospital Moinhos de Vento - Porto Alegre (RS), Brazil.; 2 Universidade Federal de Ciências da Saúde de Porto Alegre Porto Alegre RS Brazil Universidade Federal de Ciências da Saúde de Porto Alegre - Porto Alegre (RS), Brazil.

**TO THE EDITOR**

We sincerely appreciate the thoughtful comments from Dr. Michael Ayad, Dr. Hannah Lorking, and Dr. Vishal Gupta regarding our article, "Predictive factors for high-flow nasal cannula failure in patients with acute viral bronchiolitis admitted to the pediatric intensive care unit".^(1)^ Their observations are highly relevant and enrich the discussion on high-flow nasal cannula (HFNC) therapy in infants with acute viral bronchiolitis (AVB).^(2)^

As highlighted by Ayad et al.,^(2)^ age is a crucial factor in bronchiolitis, as respiratory reserve and pulmonary compliance vary markedly during infancy. In our cohort of patients under 24 months (median of 4 months, interquartile range [IQR] 2 - 7), those who failed HFNC were significantly younger (2 *versus* 5 months; p = 0.001).^(1)^ Age was excluded from multivariate analysis due to strong collinearity with weight and initial flow (r > 0.8). Therefore, weight was used as a representative marker of physiological maturity and flow adjustment. Previous studies also identified younger age and lower weight as predictors of HFNC failure,^(3,4)^ consistent with our results.

We acknowledge the authors’ interest in prematurity. In our sample, 10.5% of infants (n = 17) were preterm,^(1)^ but this factor was not significantly associated with HFNC failure (p = 0.178). It is important to note that our database did not allow stratification of prematurity into extreme, moderate, or late preterm, which limits a more detailed analysis of the impact of gestational age. Although prematurity is a recognized risk factor for respiratory morbidity, its influence on HFNC outcomes may depend on subtle interactions between gestational and postnatal lung maturity. Future multicenter studies with larger samples are warranted to explore this association, particularly among extremely preterm infants.

Concerning chronic comorbidities, 13 patients had relevant conditions (5 neurological, 8 cardiac), with 12 in the success group (p > 0.05).^(1)^ These patients were retained to reflect the real-world pediatric intensive care unit (ICU) population, where comorbidities frequently coexist with viral illnesses.

We agree that viral etiology warrants attention. Respiratory syncytial virus remains particularly relevant due to its tropism for the lower airways and its association with prolonged hospitalizations. However, our retrospective design and inconsistent viral panel documentation precluded stratification by specific pathogens. Future studies incorporating standardized viral testing could better elucidate correlations between viral agents, HFNC failure, and the need for ventilatory escalation.

Three patients (1.9%) presented with mild pleural effusion, without evidence of bacterial pneumonia or heart failure.^(1)^ Although uncommon in classic viral bronchiolitis, small reactive effusions may occur due to intense viral inflammation. Differentiating reactive from early bacterial effusions is subtle and represents a study limitation. These patients were included, as there was no association with worse outcomes.

We sincerely appreciate your careful reading and insightful comments regarding the flow rates used in our study. We would like to clarify an important distinction: all patients in our cohort initiated HFNC therapy at 2L/kg/min, as per our protocol. This approach is consistent with previous studies and current clinical practice.^(5)^

The 12L/min threshold mentioned in our article was not the starting flow for all patients, but rather a cutoff value determined from ROC curves to identify patients at higher risk of HFNC failure. In our analysis, an initial flow ≤ 12L/min was the most predictive for failure (AUC = 0.71; 95%CI: 0.61 - 0.84; p = 0.001).^(1)^ While this value approximately corresponds to a patient weighing 6kg receiving 2L/kg/min, it was applied uniformly across our heterogeneous population for statistical standardization.

We recognize that factors such as age, prematurity, and comorbidities influence weight and, consequently, the absolute flow in L/min. However, using the ROC-derived cutoff of 12L/min allowed us to identify patients with a higher likelihood of failure while still following the 2L/kg/min initiation protocol. We agree that future studies with larger samples could provide additional guidance on the comparative utility of L/kg *versus* absolute L/min flow rates, particularly in children weighing less than 5kg.

In summary, younger age (and lower weight) and insufficient initial flow were key predictors of HFNC failure in infants with acute viral bronchiolitis, likely reflecting the higher metabolic demand and greater respiratory effort in smaller infants. Insufficient initial flow may fail to meet inspiratory needs, resulting in persistent respiratory effort and early fatigue.

We sincerely thank Ayad et al.^(2)^ once again for their valuable suggestions. Prospective studies are essential to further understand these mechanisms and to refine the predictive criteria for HFNC failure.
